# Genomic insights into *Bacillus subtilis* MBB3B9 mediated aluminium stress mitigation for enhanced rice growth

**DOI:** 10.1038/s41598-023-42804-9

**Published:** 2023-09-30

**Authors:** Dibya Jyoti Hazarika, Sudipta Sankar Bora, Romen Singh Naorem, Darshana Sharma, Robin Chandra Boro, Madhumita Barooah

**Affiliations:** 1 DBT - North East Centre for Agricultural Biotechnology, Assam Agricultural University, Jorhat, Assam 785013 India; 2 Department of Agricultural Biotechnology, Assam Agricultural University, Jorhat, Assam 785013 India

**Keywords:** Microbiology, Applied microbiology, Microbial genetics

## Abstract

Aluminium (Al) toxicity in acid soil ecosystems is a major impediment to crop production as it drastically affects plant root growth, thereby acquisition of nutrients from the soil. Plant growth-promoting bacteria offers an interesting avenue for promoting plant growth under an Al-phytotoxic environment. Here, we report the plant growth-promoting activities of an acid-tolerant isolate of *Bacillus subtilis* that could ameliorate acid-induced Al-stress in rice (*Oryza sativa* L.). The whole genome sequence data identified the major genes and genetic pathways in *B. subtilis* MBB3B9, which contribute to the plant growth promotion in acidic pH. Genetic pathways for organic acid production, denitrification, urea metabolism, indole-3-acetic acid (IAA) production, and cytokinin biosynthesis were identified as major genetic machinery for plant growth promotion and mitigation of Al-stress in plants. The in-vitro analyses revealed the production of siderophores and organic acid production as primary mechanisms for mitigation of Al-toxicity. Other plant growth-promoting properties such as phosphate solubilization, zinc solubilization, and IAA production were also detected in significant levels. Pot experiments involving rice under acidic pH and elevated concentrations of aluminium chloride (AlCl_3_) suggested that soil treatment with bacterial isolate MBB3B9 could enhance plant growth and productivity compared to untreated plants. A significant increase in plant growth and productivity was recorded in terms of plant height, chlorophyll content, tiller number, panicle number, grain yield, root growth, and root biomass production.

## Introduction

Soil contamination with toxic heavy metals such as lead, selenium, arsenic, aluminum, and iron is a major issue worldwide and affects a significant amount of agricultural land. In fact, it has been estimated that about 40–50% of the world's potentially arable land (more than 800 million hectares) is affected by aluminum-related acid toxicity^1,2^. Aluminum toxicity is a primary limiting factor for plant growth and development in acid soils, as it results in poor root development and stunted growth^2,3^. Aluminium (Al) is the third most abundant element (after oxygen and silicon) in the Earth’s crust. Its solubilization leading to its accumulation and speciation is largely influenced by the chemical environment and the pH of the soil. In acid soils with a pH value lower than 4.3, the metal is solubilized into the forms such as [Al(H_2_O)_6_]^3+^ (hexaaquaaluminium (III) ion), Al(OH)^2+^, Al(OH)^3−^, and Al(OH)^4−^ (hydroxide ions of aluminium), which can become toxic to plants, particularly the [Al(H_2_O)_6_]^3+^ (commonly known as the Al^3+^) form^4–6^. This toxic form of aluminum can limit plant growth and development by inhibiting the development of extensive root systems, resulting in stunt and brittle roots, poor root hairs, and swollen root apices. Although Al-toxicity in acid soils can be ameliorated through the application of agricultural limes such as limes [limestone (calcium carbonate)—CaCO_3_ and dolomite—CaMg(CO_3_)_2_] or other physical correction measures to neutralize soil acidity, the process is laborious and not very cost-effective.

The use of acid-tolerant plant growth-promoting bacteria (PGPB), especially the siderophore-producing rhizobacteria, offers an interesting avenue for promoting plant growth under an Al–Fe–phytotoxic environment. These bacteria help in mitigating the effects of aluminum toxicity in plants by promoting plant growth and inducing systemic resistance to biotic and abiotic stress. Rhizobacterial isolates viz. *Pseudomonas simiae* N3, *Chryseobacterium polytrichastri* N10 and *Burkholderia ginsengiterrae* N11-2 sourced from the rhizosphere of diseased Korean ginseng roots were able to support the growth of *Arabidopsis thaliana* stressed by Al^7^. Several bacterial species are known to produce different siderophore compounds, including bacillibactin (produced by *Bacillus subtilis*), schizokinen, N-deoxyschizokinen (produced by *Bacillus megaterium*), enterobactin, enterobactin, salmochelin, aerobactin, and yersiniabactin (produced by the members of enterobacteriaceae family)^8–10^. Apart from siderophore production, ACC deaminase activity, ammonia production, HCN production, and phosphate solubilization are some of the indirect plant-growth promoting (PGP) traits found among the bacterial isolates, which provide abiotic stress tolerance in plants^11–14^.

Over the past decade, the genome sequencing approach has been widely employed to investigate the molecular mechanism of bacteria to tolerate abiotic stresses. Here, we conducted a whole genome sequence analysis of an acid-tolerant rhizospheric bacterial isolate to understand the mechanisms of Al-tolerance and plant growth promotion in acidic pH. We also demonstrate the efficiency of the bacterial isolate to mitigate acidic pH-induced Al-toxicity in rice plants.

## Results

### Characterization of the acid tolerant bacterial isolate

In the present study, a bacterial isolate (designated as MBB3B9) obtained from the rhizosphere of rice grown in acidic soil was characterized to evaluate its ability to tolerate acidic pH. Preliminary investigations on growth analysis at different pH suggested that the bacterial isolate MBB3B9 was able to tolerate acidic pH of 4.5 and more, while no growth was observed at pH 4.0 and 3.5 (Supplementary Fig. S1). Based on the morphological, biochemical, and 16S rRNA gene sequence analysis, the identity of this bacterial isolate was revealed as *Bacillus subtilis* MBB3B9 (Supplementary Table S1). The 16S rRNA gene sequencing was performed to define the strain taxonomy, finding that it belongs to the *Bacillus subtilis* species with a query cover of 100%, an identity of 99%, and an E-value of 0.0.

### Determination of Al-tolerance in* B. subtilis* MBB3B9

**Figure 1:**
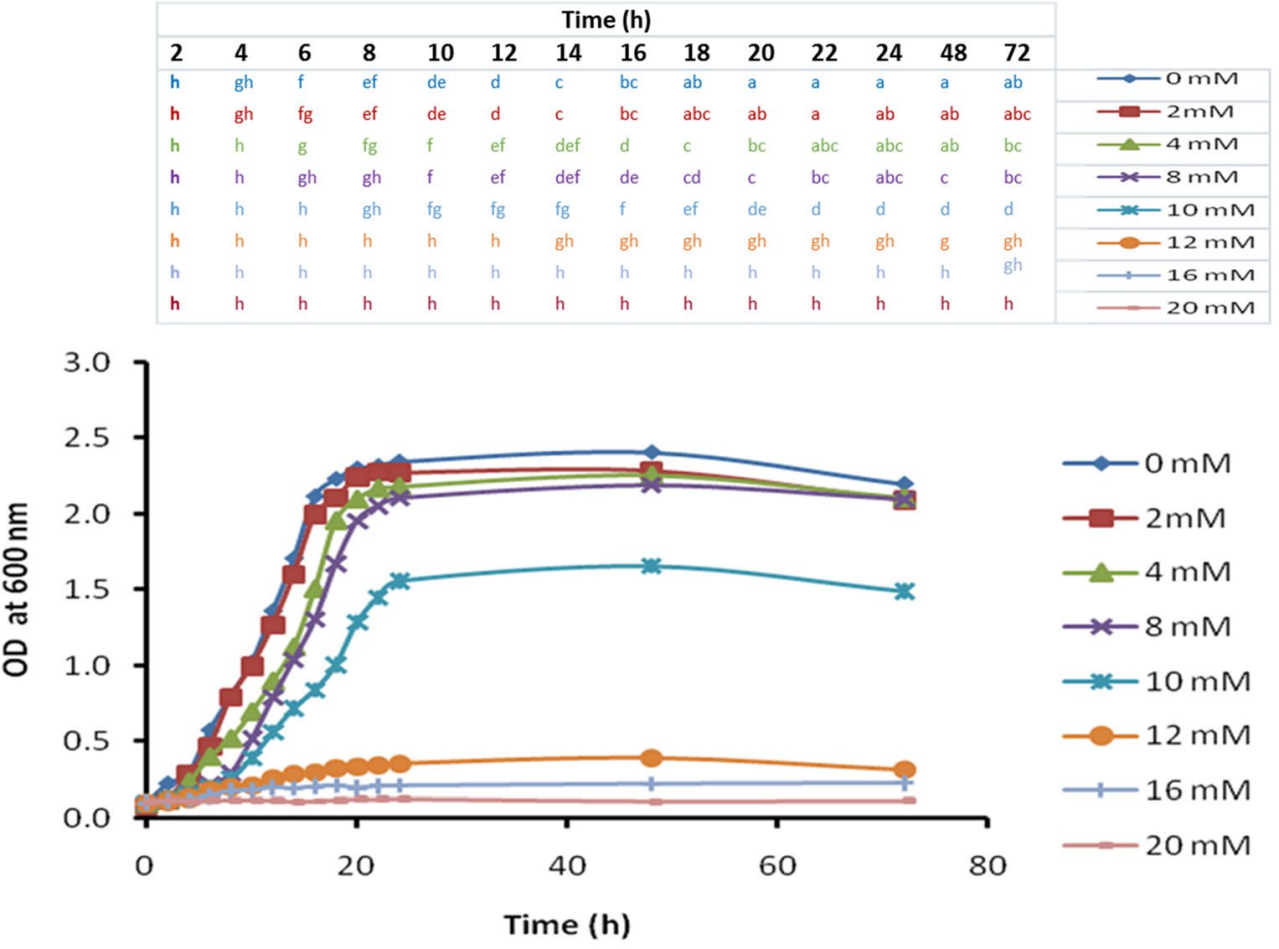
Growth curve analysis of isolate MBB3B9 in nutrient broth supplemented with different concentrations of AlCl_3_. Lower value for OD_600_ represents lower cell density. Statistical significance calculated using one-way ANOVA and Duncan’s multiple range test is shown above the graph (designated using different alphabets) for every time points with different color codes.

Al-tolerance in *B. subtilis* MBB3B9 was assessed in nutrient broth (NB) containing varying concentrations of AlCl_3_ (viz. 0 mM, 2 mM, 4 mM, 8 mM, 10 mM, 12 mM, 16 mM, and 20 mM), which revealed that the bacterium was able to tolerate up to 10 mM AlCl_3_ at pH 4.5. The toxic effect of aluminium was observed after incubation at an AlCl_3_ concentration of 12 mM or more, which was indicated by decreased viability of cells (Fig. 1).

### Whole genome sequencing and sequence analysis

#### Assembly and annotation

**Figure 2:**
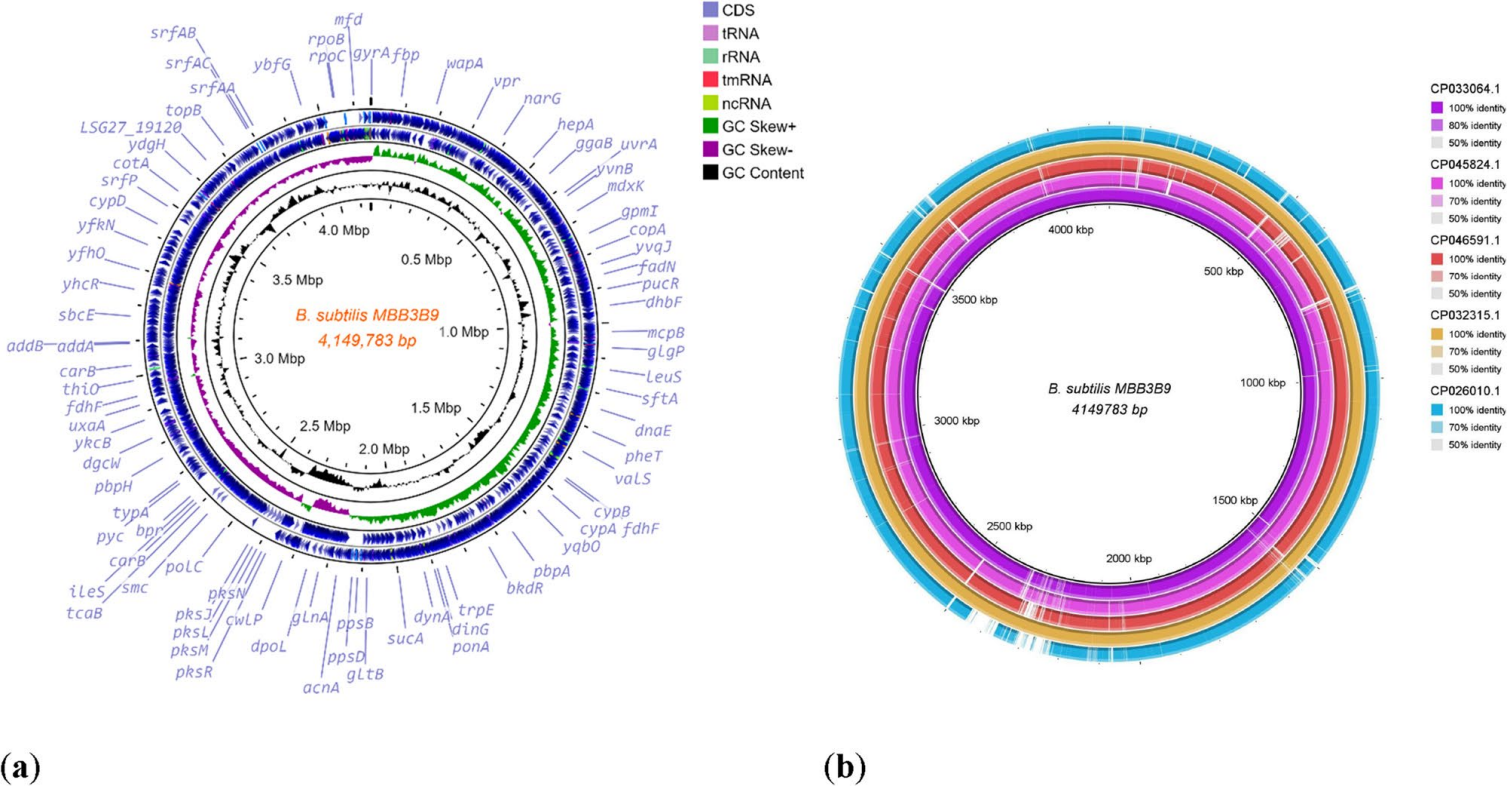
(**a**) Circular visualization of the genome map of *Bacillus subtilis* MBB3B9; (**b**) Comparative genome analysis of *Bacillus subtilis* MBB3B9 with the reference genomes.

The genome of *Bacillus subtilis* MBB3B9 was sequenced using the Illumina Truseq platform, resulting in 4,797,551 paired-end reads with coverage of 35x. The closely related strains identified by the KmerFinder 3.2 tool were *Bacillus* sp. WR11 (CP033064.1), *B. subtilis* MB8_B10 (CP045824.1), and *B. subtilis* R31 (CP046591.1). Further, the MiGA (Microbial Genomes Atlas) tool revealed that the assembled genome sequence has very high (99.1%) genome completeness, and was closely related to *B. subtilis* MZK05 (CP032315.1) with an Average Nucleotide Identity (ANI) of 99.94%. The assembled genome contigs were scaffolded into a single circular contig using the reference sequence, *B. subtilis* MZK05 (CP032315.1). The scaffolded *B. subtilis* MBB3B9 chromosome has 4,149,783 bp with 43.42% GC contents, and its annotation resulted in 4149 protein-coding sequences, 86 tRNA sequences, 14 rRNA operons, and no CRISPR and plasmid sequences (Table 1). The comparative genomes map revealed that *B. subtilis* MBB3B9 genome showed the closest similarity with B. *subtilis* MZK05 (CP032315.1), and *Bacillus* sp. WR11 (CP033064.1). While *B. subtilis* strain ATCC 11774 (CP026010.1) genome was found to contain many gaps during comparative genome analysis indicating that this strain was distinct from our study genome of *B. subtilis* MBB3B9 strain (Fig. 2).

**Table 1:** Statistics of the genome assembly.

Sl. No.	Attributes	Response
1	Number of contigs	57
2	Number of scaffold	1
3	Genome length (bp)	4,149,783
4	GC content (%)	43.42
5	CDS	4149
6	rRNA	14
7	tRNA	86

**Figure 3:**
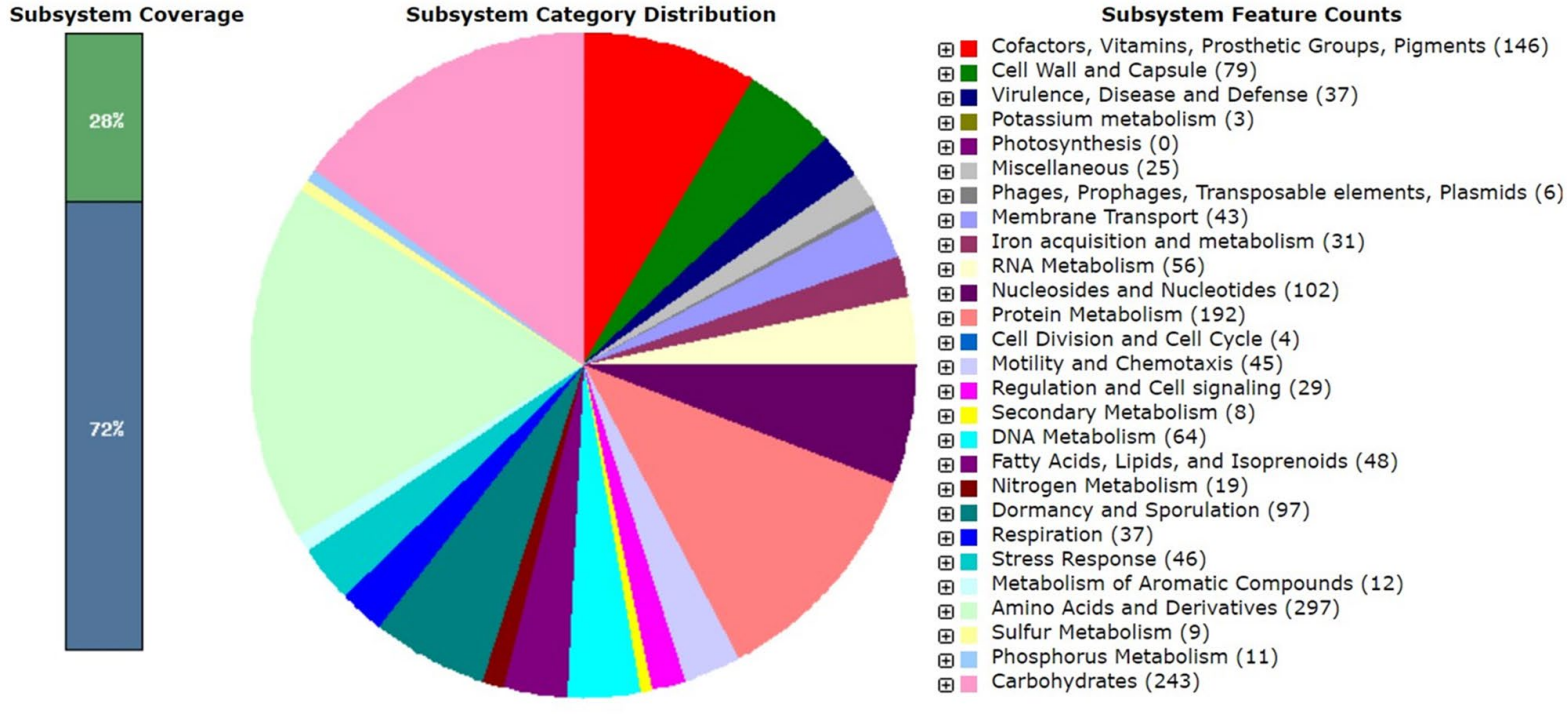
Functional annotation of the genes determined using RAST-SEED viewer.

The functional annotation of *B. subtilis* MBB3B9 genome using RAST-SEED viewer predicted that 28% of the functional proteins were in the subsystem of which 1137 CDS (coding sequence) were non-hypotheticals, and 61 CDS were hypothetical proteins. While 72% of functional proteins were not in the subsystem (Fig. 3). The functional categorization of *B. subtilis* MBB3B9 showed that amino acids and derivatives (17.58%) were the highest functional category followed by carbohydrates and protein metabolism, while potassium metabolism (0.18%) was the lowest functional category. The SEED-viewer identified 8 CDS that are involved in secondary metabolism such as lanthionine synthetase, alkylpyrane synthase, and auxin biosynthesis. The genes that encode for prophage elements, and siderophores (bacillibactin and anthrachelin) were identified. Further, several genes involved in oxidative stress response were identified in the genome of *B. subtilis* MBB3B9 including protection of reactive oxygen species, oxidative stress, bacitracin stress, and glutathione (non-redox and redox reactions).

Comparative genome analysis was carried out to identify the virulence properties of *B. subtilis* MBB3B9, which suggested that there were no genetic machineries that has a potential risk to human. Furthermore, no genes were detected which could provide multi-drug resistance to the bacterium. Comparative genome mining of *B. subtilis* MBB3B9 using virulence factor database (VFDB) and Victors program identified 12 potential genetic loci encoding virulence factors related to endopeptidase activity, transcriptional regulation, protease activity, type 7 secretion system (T7SS), surface adhesion, or bacterial imunity (Supplementary Table S3). These virulence factors (except BsrG) are also detected in the genome of the closest relative strain *B. subtilis* MZK05.

#### Phylogenetic analysis

**Figure 4:**
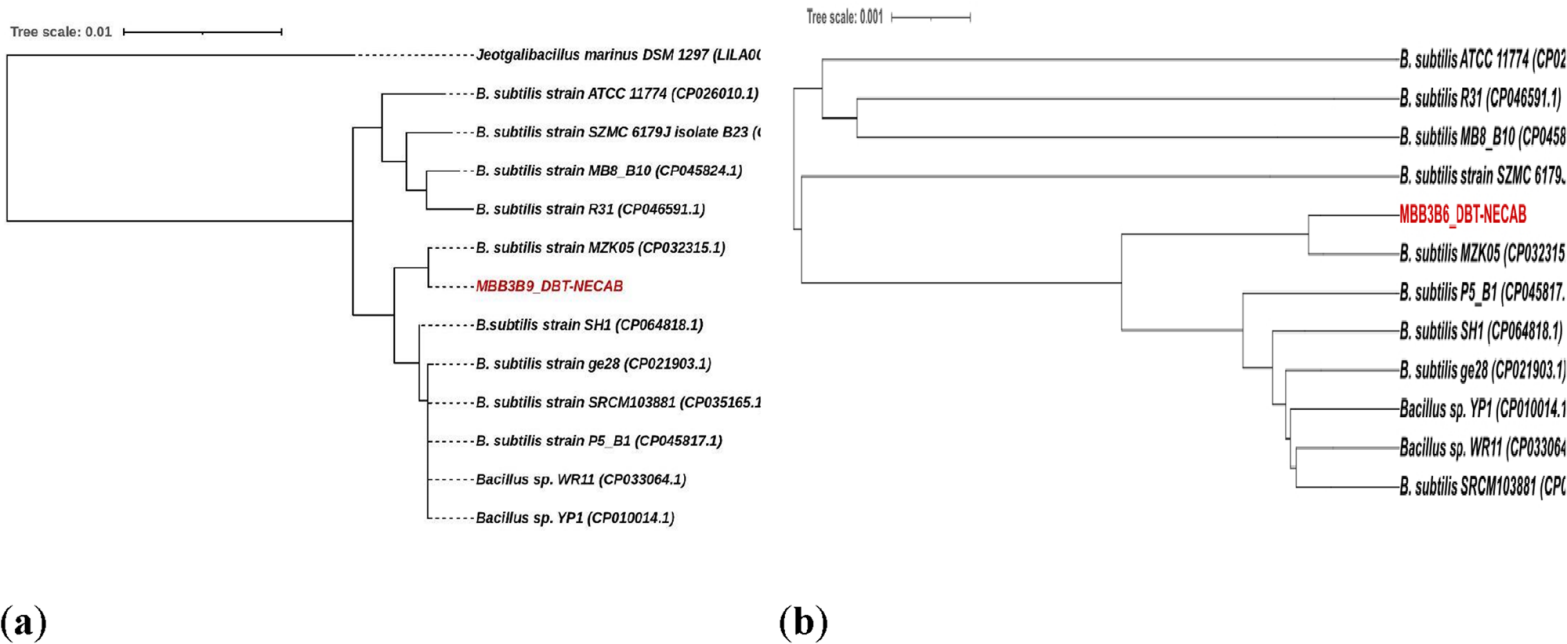
Phylogenetic analysis of *Bacillus subtilis* MBB3B9 with selected reference strains. (**a**) Multi-locus sequence typing based phylogenetic analysis of the bacterial isolate MBB3B9; (**b**) Whole genome based phylogenetic analysis of the bacterial isolate MBB3B9.

The Multilocus Sequence Typing (MLST) analysis of *B. subtilis* MBB3B9 and 11 *B. subtilis* reference strains showed that the study *B. subtilis* MBB3B9 strain possesses the unknown Sequence Type (ST), and found its nearest ST is ST-223. The majority of the reference strains belonged to ST-145. The phylogenetic tree generated from the MLST gene dataset produced three clusters, in which the strains belonging to different types of STs were found in cluster-I. In clade III, 6 strains were found clustering with those that belonged to the same ST-145. Our isolate *B. subtilis* MBB3B9 was found to form a cluster with *B. subtilis* MZK05 strain belonging to ST-223 (Fig. 4a). The MLST analysis of *B. subtilis* MBB3B9 predicted that the ST is unknown, and its nearest ST is ST-223. The ANI % and comparative genome map studied confirmed that these two strains were in proximity to each other. The MLST tree is reported to provide high resolution in terms of clustering similar or closer bacterial strains and therefore, a whole genome phylogenetic tree was also constructed using the same parameters for comparative analysis (Fig. 4b). The analysis revealed both the constructed trees to form three major clusters (excluding the outgroup taxa in the MLST tree), and MBB3B9 grouped with the reference strain MZK05 in both cases.

#### Prediction of metabolic pathways for Al-tolerance and PGP activities

Genome-wide analysis suggested the presence of genes for the production of siderophores that could potentially be involved in chelating metal ions. Biosynthetic gene clusters to produce bacillibactin and enterobactin, and their trans-membrane transportation were detected in the genome of *B. subtilis* MBB3B9 (Table 2, Supplementary Table S2). Several plant growth promotion-related genes were also detected, which contributed to the solubilization of phosphate and potassium sources, fixation and biotransformation of different nitrogen forms, and production of phytohormones like auxin and cytokinin. Some of the important genes and their functional properties that contributed to plant growth promotion are presented in Table 2. Details of the enzymes and their KEGG annotations are presented in Supplementary Table S2. Major biochemical pathways included the biosynthetic pathways for various organic acids such as citric acid, acetic acid, malic acid, gluconic acid, oxalic acid, oxaloacetic acid, succinic acid, malonic acid, glycolic acid, lactic acid, etc. Genes such as *phoA*, *phoD* and *phoE* (with phosphatase activity), *phnO* (from the phosphonate degradation pathway), *ppx*, *phtA*, *phtB*, and *phtC* (phosphate transporters), etc. were detected in the genome of *B. subtilis* MBB3B9 (Table 2, Supplementary Table S2). The presence of several nitrogen metabolism-related genes including those for nitrogenase biosynthesis, denitrification, ammonia production from glutamate-glutamine interconversion, urea metabolism, and transport of glutamine and ammonia were also identified, which indicated the active role of this bacterial isolate in plant growth promotion (Table 2, Supplementary Table S2).

**Table 2:** Plant growth promoting genes and their respective functions related to Al-stress mitigation under acidic pH.

PGP properties	Gene	Particular function
Siderophore production	*dhbA*, *dhbB*, *dhbC*, *dhbE*, *dhbF*	Bacillibactin biosynthesis
	*ymfD*	Bacillibactin exporter
	*ghrB*	Keto gluconate biosynthesis
	*maeA/sfcA*	Malic/pyruvic acid biosynthesis
	*ackA*	Propionic acid biosynthesis
	*pta, acyP, actP*	Acetic acid biosynthesis
	*lpd/pdhD, aceF/pdhC*	Acetoin 2,3-butandiol biosynthesis
Phosphate/potassium solubilizaation	*pckA, pyc*	Oxaloacetic acid biosynthesis
	*mdh, fumC*	Malic acid biosynthesis
	*frdA, sucA, sucB, sucC, sucD, prpB, dctA, fadA/fadI, sdhA/frdA*	Succinic acid biosynthesis/transport
	*CS, citM, citS*	Citric acid biosynthesis/transport
	*fadA/fadI*	Succinic/jasmonic acid biosynthesis
	*gabD, sdhB/frdB, sdhC/frdC*	Fumaric acid biosynthesis
	*accA, accB, accC, accD, pccB*	Malonic acid biosynthesis
	*ldh, gloB/gloC, mgsA, lctP*	Lactic acid Biosynthesis/transport
	*ptb, buk*	Butyric acid biosynthesis
	*ybgC*	Valeric acid biosynthesis
	*ilvD, dat, tpa, ilvA/tdcB, dsdA, sdaA/sdaB/tdcG, patB, pyk*	Pyruvic acid biosynthesis
	*pta, acs, acuI, mmsA/iolA, mmdA*	Propionic acid biosynthesis
	*ttuB*	Tartaric acid transport
	*phnO*	Phosphonate degradation
	*phoA, phoD, phoE*	Phosphatase activity
	*phy*	Phytase production
	*pstA, pstB, pstC*	Phosphate transport
	*nifF, nifU*	Nitrogenase biosynthesis
	*narG/narZ/nxrA, nfrA1, nfrA2/ycnD, narK/nrtP/nrt/narU, norQ, nasA, nirB, narH/narY/nxrB, narI/narV, narJ/narW, nirC*	Denitrification
Nitrogen metabolism	*gltA, gltB, gltD, glnA, glnB*	Glutamate/glutamine metabolism
	*gltP/gltT, peb1A/glnH, peb1C/glnQ, peb1B/glnP/glnM, glnK, glnL, glnT*	Glutamate/glutamine transport
	*ureA, ureB, ureC*	Urea metabolism
Auxin production	*pyc*, *bsdC*, *aldH*, *patA*, *poxL*, *yedL*	Indole 3-acetic acid biosynthesis
	*trpA*, *trpB*, *trpC*, *trpD*, *trpE*	Tryptophan biosynthesis
Cytokinin production	*miaA/ipt, miaB, dapF*	Cytokinin biosynthesis
	*xdhA, xdhB, xdhC*	Xanthine biosynthesis

Biochemical pathways for tryptophan biosynthesis (genes of the *trp* operon) and indole 3-acetic acid biosynthesis were detected in the genome (Table 2, Supplementary Table S2), suggesting the active role of these genes in root elongation. Similarly, the ability to produce cytokinin—another plant growth hormone important for shoot elongation, was evidenced by the presence of cytokinin biosynthetic genes in the genome of *B. subtilis* MBB3B9 (Table 2, Supplementary Table S2).

**Figure 5:**
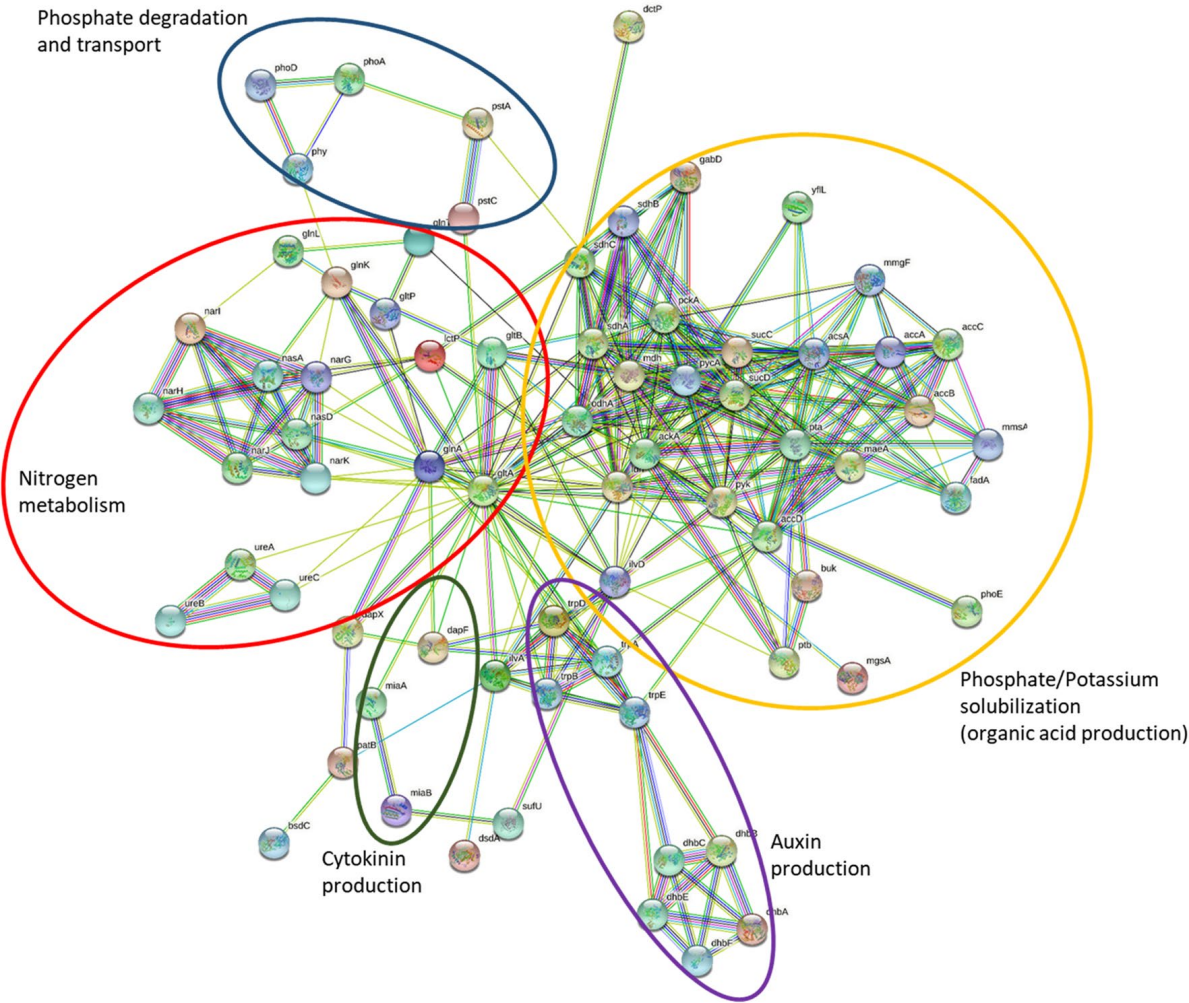
Interaction network of the plant growth promotion trait associated genes in *Bacillus subtilis* MBB3B9.

In silico protein–protein interaction analysis was performed with selected PGP-related genes using String (http://string-db.org/), which suggested strong relationships among the genes with specific functions such as nitrogen metabolism, phosphate/potassium solubilization, phytohormone production, etc. Some of the selected genes such as *gltA*, *gltB*, *glnA*, *odhA*, *ilvD*, etc. showed strong interactions with other genes from different functional properties, thus acting as bridges among multiple PGP functions (Fig. 5).

#### Prediction of secondary metabolite biosynthetic genes

**Figure 6:**
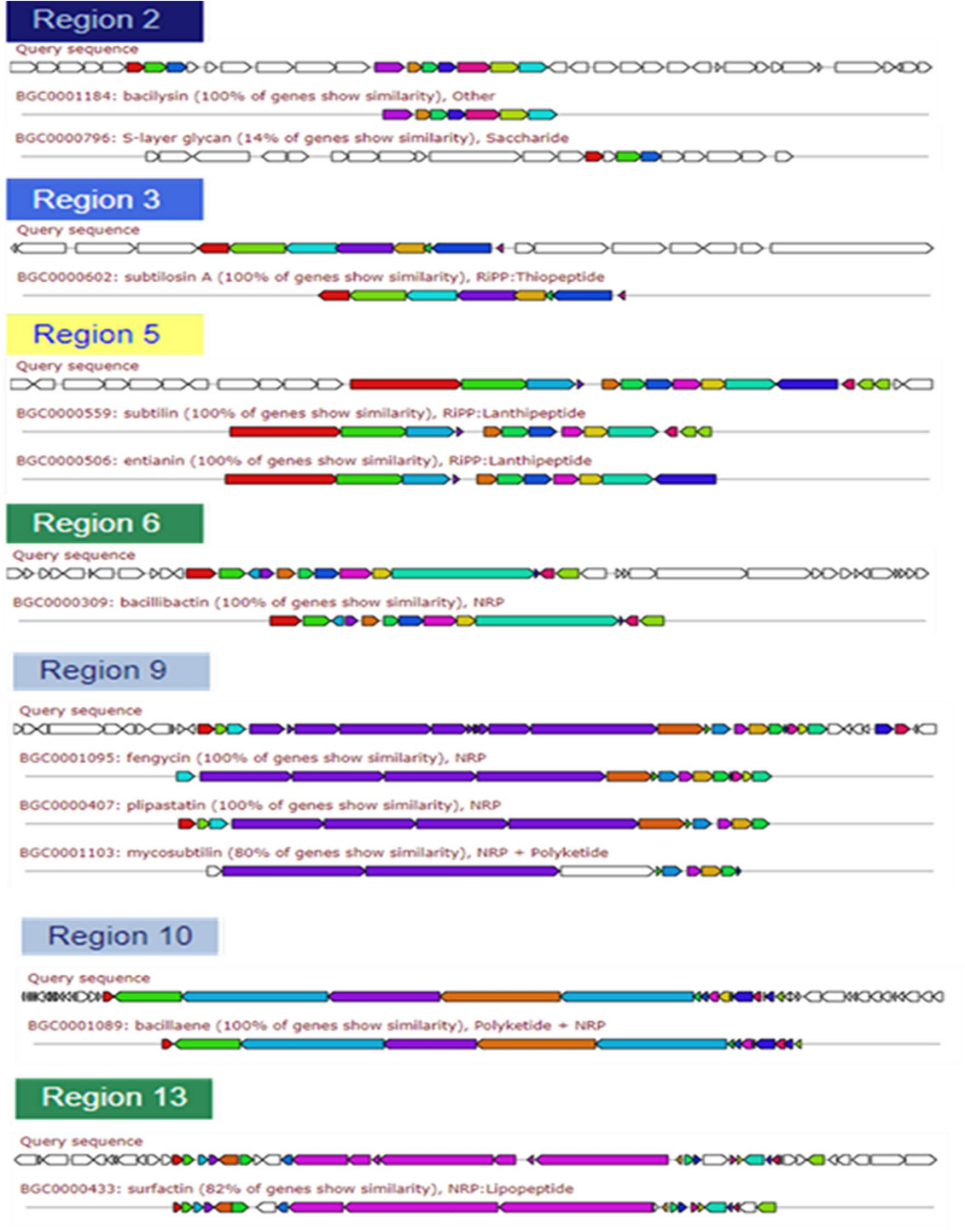
Secondary metabolites biosynthetic gene clusters detected in *B. subtilis* MBB3B9 genome.

Secondary metabolite biosynthetic gene cluster prediction using the antiSMASH web tool indicated the presence of 13 regions containing the gene clusters involved in various secondary metabolites production. Among those 13 clusters, 6 gene clusters (regions: 2, 3 5, 6, 9, and 10) showed 100% similarity of genes to the biosynthetic gene-clusters of bacilysin, subtilosin A, subtilin/entianin, bacillibactin, fengycin/plipastatin, and bacillaene, respectively. About 80% of genes in region 10 and 82% of genes in region 13 showed similarity to mycosubtilin and surfactin biosynthesis, respectively (Fig. 6). Other gene clusters were also detected to contain genes for thailanstatin A (10% of genes in region 1 showed similarity), tRNA-dependent cyclodipeptide synthases (region 4), type III polyketide synthases (region 7), terpene biosynthesis (regions 8 and 12), and lanthipeptide (class I) biosynthesis (region 11). Among the NRPS biosynthesis gene clusters, coding regions for fengycin and surfactin biosynthesis were detected, but no gene cluster was detected for iturin biosynthesis. Our findings suggested the secondary metabolites biosynthesis potential of *B. subtilis* MBB3B9, which may impart advantages to interspecific competitions among bacterial and fungal pathogens in the rhizosphere. Plant growth promoting properties of *B. subtilis* MBB3B9. Comparative genome analysis has suggested that these gene clusters were also present in the genome of *B. subtilis* MZK05 and a few other close relatives of *B. subtilis* MBB3B9, and 100% of the genes within those 7 gene clusters showed similarities to the reference gene clusters (data not shown).

### In-vitro plant growth properties of *B. subtilis* MBB3B9

In the present study, siderophores production was assessed through the Chrome Azurol S (CAS) assay. The results suggested that isolate MBB3B9 was a good siderophore producer (Table 3). The bacterium was also able to show a high PSI value (3.17 ± 0.39) for solubilizing tricalcium phosphate suggesting its ability to convert insoluble phosphates (especially, tricalcium phosphate) to their available forms that are suitable for plant uptake. On the other hand, the bacterium showed poor phosphate solubilization efficiency when aluminium phosphate was used as substrate. Similarly, the bacterial isolate also showed high activity for the solubilization of zinc (ZSI = 2.96 ± 0.28) when zinc oxide was given as substrate.

**Table 3:** Plant growth promoting properties of *B. subtilis* MBB3B9.

Sl. No.	Attributes	Response
1	Maximum Al tolerance	10 mM
2	Siderophore index	2.13 ± 0.51
3	% siderophore units	17.16 ± 1.65
4	IAA production (μg/mL)	48.78 ± 0.63 (day 3); 62.85 ± 1.84 (day 7)
5	Phosphate solubilization (PSI)	3.17 ± 0.19 (Tricalcium phosphate); not detected for Aluminium phosphate
6	Zinc solubilization index (ZSI)	2.96 ± 0.28

The development of red color indicated IAA production from tryptophan supplemented to the culture medium of *B. subtilis* MBB3B9. From the calibration curve prepared using the standard IAA solutions of known concentrations, the IAA production was determined as 48.78 ± 0.63 µg/mL and 62.85 ± 1.84 µg/mL after 3 and 7 days of inoculation, respectively (Table 3).

### Endophytic colonization assay

**Figure 7:**
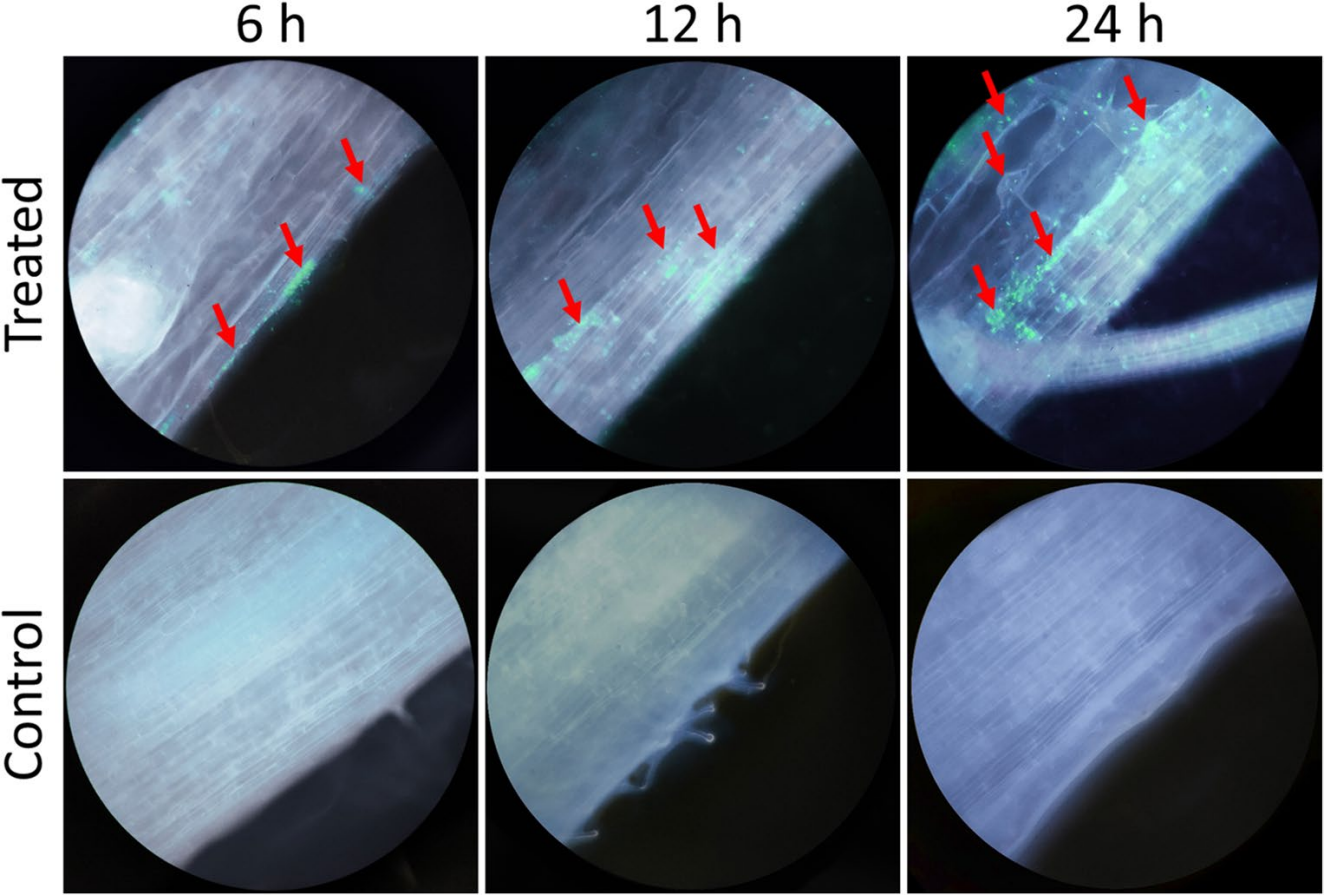
Colonization of *Bacillus subtilis* MBB3B9(GFPuv^+^) inside the root tissue of rice. Red arrows indicate the presence of green fluorescence suggesting the colonization of GFP-tagged bacterial cells.

The ability of *Bacillus subtilis* MBB3B9 to colonize the rice root was assessed by inoculating the rice roots with GFP (green fluorescent protein)-tagged bacterial cells [*Bacillus subtilis* MBB3B9(GFPuv^+^)] and observing under fluorescence microscopy. The GFP-tagged cells emitted a constant fluorescence upon IPTG (isopropyl β-D-1-thiogalactopyranoside) treatment allowing easy differentiation from the background auto-fluorescence of the root tissue (Fig. 7). In contrast, the samples inoculated with wild-type cells as well as the samples without any bacterial treatment did not produce the typical green fluorescence. The fluorescence microscopy images confirmed that during the initial stages (6 h of inoculation) the GFP-tagged bacterial cells colonized over the root surface, while at the later stages (12–24 of inoculation) bacterial cells were observed along the internal parts of the root tissues. Cells were detected on the root surface as single cells or clustered forms (Fig. 7).

### Assessment of Al-stress amelioration and plant growth promotion

Growth characteristics of the treated and untreated rice plants (variety *Luit*) recorded tillering and harvesting stages are presented in Tables 4 and 5. With increasing concentrations of AlCl_3_, there were significant differences in the plant height, tiller number, and chlorophyll content at the maximum tillering stage in untreated samples. It was clearly observed that treatment with MBB3B9 significantly increased the plant height, tiller number, and chlorophyll content compared to the untreated plants under Al-stress (Supplementary Fig. S2; Tables 4 and 5). The effects of Al-stress were further observed in the harvesting stage in terms of panicle number, grain per pinnacle, and grain quality. Overall yield was reduced in the bacterium-untreated plants compared to the bacterium-treated plants grown under increased concentrations of AlCl_3_ (Table 5).

**Table 4:** Growth parameters of the bacterium-treated (T) and untreated (UT) rice plants at various Al-stress conditions after 60 days of transplanting (maximum tillering stage). Data are represented as the mean ± standard error of three independent replications. Different letters (superscripted) in each row indicate that the results for each parameters are significantly different, otherwise insignificant.

Parameters	pH 6.8 Control		pH 4.6 Control		pH 4.6 (+ 25 µM AlCl_3_)		pH 4.6 (+ 50 µM AlCl_3_)		pH 4.6 (+ 100 µM AlCl_3_)	
	UT	T	UT	T	UT	T	UT	T	UT	T
Plant height (cm)	60.3 ± 2.3^b^	67.7 ± 1.6^a^	51.8 ± 2.5^c^	63.6 ± 2.1^ab^	51.3 ± 1.6^c^	63.8 ± 2.1^ab^	52.0 ± 1.7^c^	58.9 ± 2.9^b^	48.2 ± 1.4^c^	58.3 ± 2.2^b^
Tiller number	6.33 ± 0.29^ab^	7.00 ± 0.44^a^	5.00 ± 0.29^bcd^	6.11 ± 0.35^ab^	4.44 ± 0.56^cde^	5.78 ± 0.36^abc^	3.78 ± 0.40^de^	5.33 ± 0.65^bc^	3.56 ± 0.34^e^	5.22 ± 0.46^bc^
Chlorophyll content (mg/g)	3.46 ± 0.14^bc^	3.92 ± 0.11^a^	3.36 ± 0.09^c^	3.83 ± 0.11^ab^	2.90 ± 0.19^d^	3.66 ± 0.16^abc^	2.52 ± 0.13^e^	3.48 ± 0.11^bc^	2.21 ± 0.12^e^	2.91 ± 0.09^d^

**Table 5:** Growth parameters of the bacterium-treated (T) and untreated (UT) rice plants at various Al-stress conditions after 90 days of transplanting (harvesting stage). Data are represented as the mean ± standard error of three independent replications. Different letters (superscripted) in each row indicate that the results for each parameters are significantly different, otherwise insignificant.

Parameters	pH 6.8 Control		pH 4.6 Control		pH 4.6 (+ 25 µM AlCl_3_)		pH 4.6 (+ 50 µM AlCl_3_)		pH 4.6 (+ 100 µM AlCl_3_)	
	UT	T	UT	T	UT	T	UT	T	UT	T
Plant height (cm)	83.44 ± 1.48^ab^	86.56 ± 1.47^a^	78.56 ± 1.55^bc^	83.22 ± 2.44^ab^	71.78 ± 1.51^d^	78.78 ± 2.29^bc^	69.56 ± 2.19^d^	75.89 ± 2.29^ cd^	60.56 ± 3.22^e^	73.33 ± 2.39^ cd^
Tiller number	83.44 ± 1.48^ab^	86.56 ± 1.47^a^	78.56 ± 1.55^bc^	83.22 ± 2.44^ab^	71.78 ± 1.51^d^	78.78 ± 2.29^bc^	69.56 ± 2.19^d^	75.89 ± 2.29^ cd^	60.56 ± 3.22^e^	73.33 ± 2.39^ cd^
Chlorophyll content (mg/g)	83.44 ± 1.48^ab^	86.56 ± 1.47^a^	78.56 ± 1.55^bc^	83.22 ± 2.44^ab^	71.78 ± 1.51^d^	78.78 ± 2.29^bc^	69.56 ± 2.19^d^	75.89 ± 2.29^ cd^	60.56 ± 3.22^e^	73.33 ± 2.39^ cd^
Number of panicles	4.44 ± 0.24^ab^	5.00 ± 0.28^a^	4.11 ± 0.26^ab^	4.78 ± 0.32^a^	3.56 ± 0.41^bc^	4.67 ± 0.33^a^	2.67 ± 0.24^ cd^	4.22 ± 0.46^ab^	1.89 ± 0.26^d^	4.00 ± 0.41^ab^
Yield (g/plant)	14.02 ± 1.24^ab^	15.64 ± 1.35^a^	12.06 ± 0.88^ab^	14.89 ± 1.01^a^	10.80 ± 1.23^bc^	14.41 ± 1.26	8.22 ± 0.89^ cd^	13.10 ± 1.22^ab^	5.26 ± 0.75^d^	12.67 ± 1.28^ab^
1000 seed weight	22.00 ± 0.11^a^	22.10 ± 0.19^a^	21.80 ± 0.08^a^	22.24 ± 0.20^a^	21.84 ± 0.16^a^	22.16 ± 0.25^a^	20.84 ± 0.28^bc^	21.07 ± 0.30^b^	20.03 ± 0.26^d^	20.40 ± 0.14^ cd^
Root length (cm)	25.17 ± 1.47^ab^	27.26 ± 1.93^a^	22.09 ± 1.49^bc^	25.67 ± 2.00^bc^	21.48 ± 1.36^bc^	24.96 ± 1.23^ab^	17.59 ± 1.03^de^	22.54 ± 1.19^bc^	15.17 ± 1.35^e^	20.58 ± 1.10^ cd^
Root biomass (g)	4.77 ± 0.25^ab^	5.06 ± 0.27^a^	4.23 ± 0.28^bc^	4.30 ± 0.13^bc^	3.85 ± 0.23^c^	4.27 ± 0.15^bc^	3.21 ± 0.16^d^	4.12 ± 0.20^c^	2.76 ± 0.14^d^	3.91 ± 0.12^c^

## Discussion

The current global issues, among which abiotic stress conditions represent the most important constraints on agricultural production, have led to the choice of plant–microbe systems. The beneficial effects of plant rhizospheric bacterial isolates on roots and overall plant growth are well-documented^12,15^. Among the various plant growth-promoting bacteria, *Bacillus subtilis* is one of the most extensively studied rhizobacterial species. Several studies have demonstrated the rhizospheric and endophytic colonization of *Bacillus subtilis*, thereby providing plant growth promotion and protection against various biotic and abiotic stresses^12,15–17^. Previously, Goswami et al.^18^ characterized *Bacillus subtilis* and a few other members of the genus *Bacillus* as dominant PGP bacterial taxa in the acidic soil of Assam, India. *Bacillus subtilis* and several other bacterial isolates from the genus *Bacillus* have been found associated with rice (*Oryza sativa* L.) plants^19^. *Bacillus subtilis* MBB3B9—novel plant growth-promoting bacterium isolated from acidic soil of Assam showed its ability to withstand acidic pH and showed remarkable tolerance to Al-stress. Aluminium-resistant bacterial strains from the genera *Pseudomonas*, *Burkholderia*, and *Chryseobacterium* have been reported previously by other researchers^7,20–22^.

Whole genome sequencing using Illumina platform combined with bioinformatics analysis provided insights into the general genomic features as well as detailed molecular functions of the genes in *Bacillus subtilis* MBB3B9. Bioinformatics tools such as KmerFinder and MiBG helped in the genome assembly and finding the genome completeness. Further, functional annotation of the MBB3B9 genome suggested the presence of secondary metabolism related genes (such as bacteriocins, lipopeptides, and antibiotics production gene clusters, lanthionine synthetase, alkylpyrane synthase, and phytohormone biosynthesis related genes). Many previously studied genomes of the PGP isolates of *Bacillus* spp. also harbored such gene clusters^23–25^. The genes involved in oxidative stress response provides protection of the bacterial cells from reactive oxygen species, oxidative stress, and heavy metal tolerance^26^. Comparative genome sequence data showed the highest closeness of the isolate with *Bacillus subtilis* MZK05—an industrially important strain that exhibit serine protease activity and bacteriocin production^27^. An MLST-based phylogenetic analysis validated the results of comparative genome analysis. MLST-based phylogenetic analysis approach can be employed as a highly discriminatory technique for phylogenetic analysis^28^, and can be applied for evolutionary studies and population genetics^29^. A comparison of the whole genome phylogeny and MLST-based phylogeny of *Bacillus subtilis* MBB3B9 suggested that the MLST-based phylogenetic analysis approach is an efficient technique for species identification.

Plants’ tolerance to Aluminium is directly linked to the secretion of organic acids, as well as nitrogen availability, rhizospheric production of ammonia, siderophores, and enzymes such as ACC deaminase^30–32^. Bacterial production of such enzymes and metabolites helps the plants to tolerate stress conditions. Previous studies also reported that these metabolites are produced by some strains of *Bacillus subtilis* for iron transport^9,33,34^. Resistance to Al toxicity could be correlated to the production of the siderophores^7,20^. In the present study, the isolate MBB3B9 was recorded as a good siderophore producer and the results were comparable to that of earlier findings^32,35–37^. High siderophore production by *B. subtilis* MBB3B9 indicated that the tolerance of the bacterium to the high concentration of Al is due to the production of siderophores. The *dhbACEBF* gene-cluster detected in the genome of *B. subtilis* MBB3B9 encodes the necessary enzymes to produce bacillibactin and its precursor 2,3- dihydroxybenzoate (DHB)^9,38,39^. Extracellular secretion of bacillibactin is facilitated by YmfE transporter^40^, which was also detected in the genome of *B. subtilis* MBB3B9. Various bacterial species produce several other types of siderophores, including carboxylate, catecholate, hydroxamate, and salicylate^31^. These siderophore compounds play a crucial role in the Fe and Al accumulation in the form of various organic complexes^7,41^.

A detailed investigation of its genome sequence revealed that the bacterium harbored multiple plant growth-promoting and stress-responsive pathway enzymes, along with the capability to synthesize several bioactive secondary metabolites that could show antagonistic activity against plant pathogenic bacteria and fungi. *Bacillus subtilis* and several other PGPB can solubilize phosphorus by producing various organic acids that convert the insoluble form of phosphorus into a soluble form^32,42^. Organic acids such as citric acid, oxalic acid, and malic acid secreted from the plant roots also play a vital role in the protection of plants to reduce Al toxicity^43^. Bacterial production of such organic acids in the rhizosphere could be beneficial for mitigating Al-toxicity in plants. Moreover, alkaline phosphatases, phytase production and transport machinery are important components for the phosphate solubilization.

The morphology of plant roots impacts nutrient uptake and plant growth by influencing root elongation along with the formation of lateral roots and root hairs^44^. Plant hormones are crucial components that regulate root morphology^23^. Genes for the production of IAA were detected in the genome of *B. subtilis* MBB3B9. Qualitative assessment of IAA production in *B. subtilis* MBB3B9 culture also suggested that the isolate was a good IAA producer. Production of IAA is one of the major plant growth-promoting activities that contribute to plant growth under Al-stress conditions^13,45^. Bacterial production of IAA in the rhizosphere or inside the root vascular tissue helps in the elongation of roots, thereby helping the plants to access nutrients from a wider area^32^. Production of IAA is reported from a large range of plant growth-promoting rhizobacteria. As the effect of Al-toxicity is primarily observed on roots, the application of IAA-producing rhizobacteria could have a profound effect on mitigating Al-stress in plants. IAA production induces plant growth, most commonly by improving root growth, thereby allowing the plants to access more nutrients from the soil^15,46,47^.

The genes related to plant growth promotion showed strong interactions among themselves. Expression of genes like glutamine synthase (encoded by *glnA*) plays a central role in nitrogen metabolism in both plants and microorganisms. It has been reported that glutamine causes rapid induction of the expression of important transcription factors associated with nitrogen metabolism and stress responses in rice roots^48^. An earlier study suggested that aluminium could activate the expression of chloroplastic glutamine synthetase in plants^49^. As eukaryotic glutamine synthetase is thought to be evolved from symbiotic bacteria^50^, the influence of aluminium on bacterial glutamine synthetase activity cannot be denied. Glutamate synthase encoded by the *gltA* and *gltB* genes also plays a crucial role in glutamate synthetase activity and nitrogen metabolism under stress conditions^51^. Protein–protein interaction studies also suggested that the genes from various organic acid production pathways exhibited strong interactions, which may coordinately contribute to the phosphate solubilization activity of the bacterium.

Genome mining using antiSMASH server suggested the capability of *B. subtilis* MBB3B9 to produce multiple antimicrobial secondary metabolites. At least seven gene clusters encoding antibacterial/antifungal metabolites were detected in the genome of *B. subtilis* MBB3B9. The reference genome of *Bacillus subtilis* MZK05, which was a close relative of *B. subtilis* MBB3B9, also harbored these 7 gene clusters for the biosyntesis of secondary metabolites. Although, several strains of *Bacillus subtilis* have been reported to produce antimicrobial secondary metabolites^17,24,52^, in most cases a particular bacterial strain may not produce all the secondary metabolites despite the presence of the corresponding gene clusters in its genome. Previous studies suggested altered metabolite production profiles in specific bacteria due to replacement or unavailability of core biosynthetic genes, nonsense mutation in the peptide biosynthetic genes, or altered gene expression in response to external conditions^53–55^. Alternate strategies can be beneficial to induce or overproduction of selective metabolites^56^.

Bacterial colonization inside plant roots plays an important role in the interaction between plants and PGPR (plant growth-promoting rhizobacteria)^57,58^. The ability of *Bacillus subtilis* MBB3B9 to colonize the rice roots, where the GFP-tagged bacterial cells were initially able to colonize over the root surface, while at the later stages, colonization in the internal root tissues suggested the endophytic colonization of the isolate. Several studies have reported the endophytic colonization ability of *Bacillus subtilis* in root tissues and root surfaces for different plants^59–61^. Successful colonization of plant roots by PGPR occurs either by active flagella-propelled swimming or by passive movement in water fluxes^61^.

The effect of Al-stress on rice in the presence or absence of the bacterial isolate MBB3B9 was evaluated in a greenhouse experiment using the short-duration rice variety *Luit*. Our findings confirmed the ability of this bacterial isolate to promote plant growth promotion in rice under acidic pH with toxic levels of aluminium. The effects of Al-stress could be observed distinctly in the roots. The roots under Al-stress showed stunted growth, and reduced secondary root hair development compared to that of control plants. The inhibition of root elongation is the most dramatic Al-toxicity symptom in plants^62^. Al can affect the constituents’ apoplast (pectin matrix)^63,64^ symplast (calmodulin)^65^, and the nucleic acid content in the root cells^66,67^. Al hinders cell division at the root apex and lateral roots, enhances the cell wall rigidity by cross-linking of pectins, and reduces DNA replication because of increased rigidity of the double helix^68,69^. Reduced root growth and secondary root hair development impacted the nutrient uptake of the plants, which in turn showed adverse effects on the above-ground parts including the chlorophyll content, tiller number, panicle development, and grain yield. In contrast to the untreated conditions, bacterial treatment could ameliorate the effects of Al-stress in rice plants. Root growth was not altered in the bacterium-treated plants despite high AlCl_3_ concentration, which suggested an active role of *B. subtilis* MBB3B9 on root architecture development under Al-stress conditions. This study established the potentiality of *B. subtilis* MBB3B9 as a strong candidate for biofertilizer, especially for crop production under acid-stress conditions. However, field trials in acidic soil would be useful to establish its efficiency on large-scale production sites.

## Methods

### Bacterial isolate and culture conditions

The bacterial isolate MBB3B9 was isolated from the rhizospheric soil of rice cultivated in an acidic environment at Instructional cum Research farm, Assam Agricultural University, Jorhat, Assam (location 26.721 N, 94.189 E). The isolate was maintained as pure-culture in nutrient agar (NA) medium with regular sub-culturing in fresh NA plates. Acid tolerance of the bacterial isolate MBB3B9 was assessed in nutrient broth (NB) adjusted to pH 7.0, 6.5, 6.0, 5.5, 5.0, 4.5, 4.0, and 3.5 with 0.1 M hydrochloric acid. The isolate was characterized based on its morphological and biochemical properties, as well as FAMEs (fatty acid methyl esters) profiling and 16S rRNA gene sequencing as per the protocol described earlier by Hazarika et al.^17^.

### Determination of Al-resistance in bacterial isolate

The effect of aluminium on the growth rate of the bacterium was monitored by inoculating it in an aluminium-enriched nutrient medium. The medium was prepared by mixing filter-sterilized aluminium chloride (AlCl_3_) solution to NB at appropriate proportions for the desired concentration (2 mM, 4 mM, 8 mM, 10 mM, 12 mM, 16 mM, and 20 mM). The final volume of the medium was 100 ml with pH adjusted to 4.5. Freshly grown bacterial suspension in saline solution (1 ml, OD_600_ = 0.5) was introduced to the Al-supplemented media and incubated at 30 °C with a continuous shaking at 150 rpm for 72 h. The same media without any bacterial inoculation was set as a control. Bacterial growth was monitored by recording the OD_600_ value at every 2 h interval throughout the exponential phase (up to 24 h) and at time points of 48 h and 72 h.

### Whole genome sequencing and sequence analysis

#### DNA preparation and sequencing

For isolation of the genomic DNA, a single colony of the bacterial isolate from a NA plate was grown overnight in Luria Barteny (LB) broth at 37 °C with continuous shaking. One millilitre of aliquot was collected from the culture and DNA isolation was performed using Nucleospin® Microbial DNA isolation kit (Macherey–Nagel, Germany) according to the manufacturer’s instructions. The quality of the genomic DNA was determined using ds (double-stranded) DNA HS (High Sensitivity) Assay Kit in Qubit 3.0 fluorometer (Thermo Fisher Scientific Inc., Waltham, MA, USA) and followed by 1% agarose gel electrophoresis. The genomic DNA was fragmented to 250 base pair size using an ME220-focused ultrasonicator (Covaris, Woburn, MA). The fragmented DNA size was analyzed using the TapeStation 2200 dsDNA high-sensitivity assay (Agilent Technologies, Santa Clara, CA). Further, the fragmented genomic DNA was used for the library preparation using the Illumina TruSeq library preparation kit (Illumina Inc., San Diego, CA, USA) according to the manufacturer’s protocols. Sequencing was performed using the MiSeq reagent kit version 2 (500 cycles) on the MiSeq system (Illumina, San Diego, CA), generating 2 × 250-bp paired-end reads.

#### Assembly and annotation

The raw reads (.fastq) files obtained from the sequencer were checked for their quality using FastQC (Andrews 2010) and to ensure the high quality of the reads (Phred score > 30), these were trimmed using Trimmomatic *v*0.35^70^ and de novo assembly was performed using SPAdes *v*3.12.0 with *k-mer* values of 21, 33, 55, 77, 99, and 127^71,72^. The assembled sequence was used to identify the closely related genomes using KmerFinder 3.2^73,74^ and MiGA (http://microbial-genomes.org/). The assembly stats and genome completeness quality were estimated using the QUEST *v*5.0.2^75^, and MiGA (http://microbial-genomes.org/). The identified *Bacillus subtilis* strain MZK05 (CP032315.1) was used as a reference guide to orient and order contigs using the Move Contigs module in Mauve 2.4.0^76^. Scaffolds were generated using reference-guide scaffolder MeDuSa^77^. The scaffolded sequence was annotated using Prokka *v*1.14.6^78^, and RAST (Rapid Annotation using Subsystem Technology) *v*2.0 tool^79^. The circular genome was generated and the comparative analysis was performed using the Proksee web server (https://proksee.ca/) and BRIG (BLAST Ring Image Generator)^80^. Prediction of virulence factors in the genome was performed with the help of virulence factor database (VFDB; http://www.mgc.ac.cn/VFs/)^81^ and Victors (http://www.phidias.us/victors/)^82^, which employs customized BLAST analysis for prediction of virulent proteins and toxins.

#### Phylogenetic analysis

Multilocus Sequence Typing (MLST) gene dataset (viz. *glpF*, *ilvD, pta, purH, pycA, rpoD*, and *tpiA*) of *Bacillus subtilis* strains were selected to generate MLST tree using automated Multi-Locus Species Tree (autoMLST) pipeline^83^, and visualized with Interactive Tool Of Life v4 (iTOL)^84^. Further, whole genome-based phylogeny was generated using TYGS (Type/Strain Genome Server)^85^, and visualized with Interactive Tool Of Life v4 (iTOL)^84^.

#### Prediction of metabolic pathways for PGP properties

In silico identification of genes involved in the amelioration of Al-stress, plant growth-promotion, and biocontrol traits were analyzed using multiple approaches such as SEED Viewer *v*2.0 tool assembled in RAST *v*2.0 online server^86^, and KAAS (KEGG Automatic Annotation Server). The genetic factors involved in the interaction of bacteria with the plants were identified using the PIFAR-Pred tool and further, the annotation of plant growth-promoting traits was done using the PGPT-Pred tool in the PLaBAse platform^87,88^.

#### Prediction of secondary metabolite biosynthetic genes

Secondary metabolites biosynthetic gene clusters in the genome sequence of isolate MBB3B9 were predicted using antiSMASH server version 6.0 (https://antismash.secondarymetabolites.org/). Default parameters were used to predict the secondary metabolites biosynthetic gene clusters. The Minimum Information about a Biosynthetic Gene cluster (MIBiG) comparison was used to compare the detected clusters with reference genomes.

### Determination of Al-stress related plant growth-promoting activities

#### Siderophore activity

A qualitative assay for siderophore production was performed through the Chrome Azurol S (CAS) assay^89^ with necessary modifications. The bacterial isolate was inoculated on the tryptic soy agar (TSA) plates and incubated at 30 °C for 24 h. The plates were then overlaid with CAS reagent supplemented with 1.5% agar and incubated at 30 °C for another 48 h. The appearance of an orange-yellow halo around the bacterial colony was considered a positive test for siderophore production. The ratio of the halo diameter and the colony diameter was calculated to obtain the siderophore index. For quantitative analysis, bacterial culture was freshly grown in NB (shaking speed of 120 rpm at 30 °C for 24 h). The culture was then centrifuged at 10,000 *g* for 15 min, after which 0.5 ml of CAS reagent and 10 μl shuttle solution (sulfosalicylic acid) were added to 0.5 ml of the supernatant. The solution was incubated for 2 h at room temperature and the absorbance of the solution was measured at 630 nm using a Spectroquant® Prove 300 spectrophotometer (Merck Millipore, Massachusetts, United States). A blank was also prepared using 0.5 ml of NB in place of the culture supernatant^37^. The siderophore activity was calculated using the following formula:

$$\mathrm{\% Siderophore\,unit}= \frac{Ar-As}{Ar} \times 100$$

where *Ar* = OD_630_ value of blank (CAS reagent) and *As* = OD_630_ value of sample.

#### Indole acetic acid (IAA) production

For IAA production, the bacterium was inoculated in LB broth amended with 5 mM tryptophan and incubated for 7 days at 28 °C at 200 rpm. To detect the presence of IAA, Salkowski reagent (150 ml concentrated H_2_SO_4_, 250 ml of distilled H_2_O, 7.5 ml 0.5 M FeCl_3_∙6H_2_O solution) was mixed with cell-free supernatant in the ratio of 4:1. Appearance of red indicating the presence of indolic compounds was considered as a positive test for IAA production. The intensity of color development was recorded using a Spectroquant® Prove 300 spectrophotometer (Merck Millipore, Massachusetts, United States) at 535 nm^90^. A standard curve of pure indole-3-acetic acid (range: 0–100 μg/mL) was used to determine the concentration of produced IAA.

#### Phosphate solubilization

Pikovaskya’s agar medium (Himedia, India) ameliorated with 0.5% (w/v) tricalcium phosphate/ aluminium phosphate was used for phosphate solubilization assay. The bacterial isolate was spot-inoculated onto this medium and incubated at 30 °C for 72 h. The development of a clear halo around the colony was considered a positive test for phosphate solubilization. The phosphate solubilizing index (PSI) was calculated by using the following formula:

$$\mathrm{PSI}= \frac{\mathrm{Colony\,diameter }+\mathrm{Halo\,zone\,diameter }}{\mathrm{Colony\,diameter}}$$

#### Zinc solubilization

For the zinc solubilization assay, the bacterial isolate was spot-inoculated onto a Zinc solubilizing medium (Himedia, India) containing 0.1% zinc oxide. The bacterial isolate was spot-inoculated at the centre of the plate and the inoculated plate was incubated at 30 °C for 7 days. The formation of the halo zone around the bacterial colony was considered as a positive test for zinc solubilization. The zinc solubilizing index (ZSI) was calculated by using the following formula^91^:

$$\mathrm{ZSI}= \frac{\mathrm{Colony\,diameter }+\mathrm{Halo\,zone\,diameter }}{\mathrm{Colony\,diameter}}$$

### Endophytic colonization assay

The endophytic colonization ability of the bacterial isolate in the root tissue of rice was assessed using fluorescence microscopy. For this, *Bacillus subtilis* MBB3B9 was tagged with the ultra-violet light-responsive green fluorescent protein (GFPuv) and inoculated to rice seedlings. Full-length *gfp* gene was amplified from the pGFPuv vector system using gene-specific primers GFPuv-XbaI-F (5′-CC*TCTAGA*ATGAGTAAAGGAGAAGAACTTTTCACTG-3′) and GFPuv-XmaI-R (5′-TA*CCCGGG*CATTATTTGTAGAGCTCATCCATGCC-3′) containing XbaI and XmaI restriction sites (shown in italics) flanking the CDS. The amplified product was cloned into the pHT01 vector system and transformed into *Bacillus subtilis* MBB3B9 as described previously by Goswami et al.^92^. Ten days old seedlings of rice (variety *Luit*) were treated with the cell suspension of GFP-tagged cells (in 0.85% NaCl solution containing 1 mM IPTG) for 12 h, after which the roots were washed with phosphate-buffered saline (PBS pH 7.2) and longitudinal sections were made using a sharp blade. Sections were observed under the 100X oil-immersion objective of an Olympus BX51 fluorescence microscope. The GFP-fluorescence was excited using the UV light filter and the intensity of green fluorescence was captured. Two control samples, one without any bacterial treatment and the other treated with wild-type (GFP untagged) bacterial cells, were processed in the same way and used for the comparison.

### Pot experiment

#### Rice variety

The rice variety used in this study was *Luit*- a short-duration rice variety (90–100 days) with white, medium bold non-glutinous grains and translucent endosperm. *Luit* is a semi-dwarf variety developed from the progeny between *Heera* × *Annada*^93^. It is suitable for both transplanting and direct seeding with sprouted seeds and can be grown even in flood-affected areas. The seeds were collected from AAU- Assam Rice Research Institute (formerly Regional Agricultural Research Station, Titabar), Jorhat, Assam (location 26.575 N, 94.183 E).

#### Soil preparation

Sixteen different representative top-soil samples (0–20 cm depth) were collected from one hectare of experimental tea plantation under the Instructional cum Research (ICR) farm (26.450 N, 94.130 E) at the Assam Agricultural University, Jorhat, Assam, India. All the dried soil samples were pooled homogeneously, and a composite sample was prepared which was further dried naturally and sieved through 2 mm mesh. Later the soil was divided into individual autoclave bags and sterilized three times before being put into plastic pots. The plastic pots used in this experiment had an upper diameter of 77 cm, a bottom diameter of 41 cm, and a height of 16 cm. Each pot contained 10 kg of soil amended with NPK (Nitrogen, Phosphorus, and Potassium) in a ratio of 40: 20: 20 kg/ha^19^ and 50 g of vermicompost. The test conditions included two controls at pH 6.8 and pH 4.6, along with the pots containing three different concentrations (viz. 25, 50, and 100 µM) of AlCl_3_. A total of six pots were used for each condition and divided into two groups: UT (bacterium untreated) and T (bacterium treated).

#### Bacterial treatment

Bacterial inoculum was prepared from an overnight grown culture of *B. subtilis* MBB3B9. Cells were harvested by centrifugation and the pellet was washed two times with sterile saline solution (0.85% sodium chloride). Finally, the cells were resuspended in sterile saline solution continuing 0.5% carboxymethylcellulose, and mixed with the soil in the respective pots (approximately 1 × 10^8^ cells per g of soil).

#### Transplantation and growth assessment

Twenty-day-old rice seedlings of equal height were picked from the nursery bed and transplanted in order that each pot contained three plants. The pots were filled with sufficient water to maintain a water level of 5 cm above the soil surface. Plant height, tiller number, and chlorophyll content were measured at two different stages, i.e., maximum tillering stage (60 days after transplantation) and harvesting stage (90 days after transplantation). Root growth and yield attributes were assessed after harvesting (at 90 days after transplantation).

### Statistical analysis

All the experiments were conducted with three independent replications. Data generated from this study are represented as mean ± standard error (wherever applicable). Significance among the treatments was tested using the one-way Analysis of Variance (ANOVA) at a 95% confidence level, along with Duncun’s multiple range test (*p* ≤ 0.05).

### Ethics approval

The authors confirm that the use of plants in the present study complies with institutional guidelines.

### Supplementary Information

Supplementary Information.

## Data Availability

Data generated in this research are provided as supplementary material with this manuscript. DNA sequence data are deposited in the NCBI GenBank database (accession number CP089269.1).
