# Supplementary material for: Genomic insights into Bacillus subtilis MBB3B9 mediated aluminium stress mitigation for enhanced rice growth
Source: Sci Rep. 2023 Sep 30;13:16467. doi: 10.1038/s41598-023-42804-9 (PMC10542363; doi:10.1038/s41598-023-42804-9)
Supplement: Supplementary file 1 — Supplementary Information. [file 41598_2023_42804_MOESM1_ESM.docx]

**Supplementary Information**

**
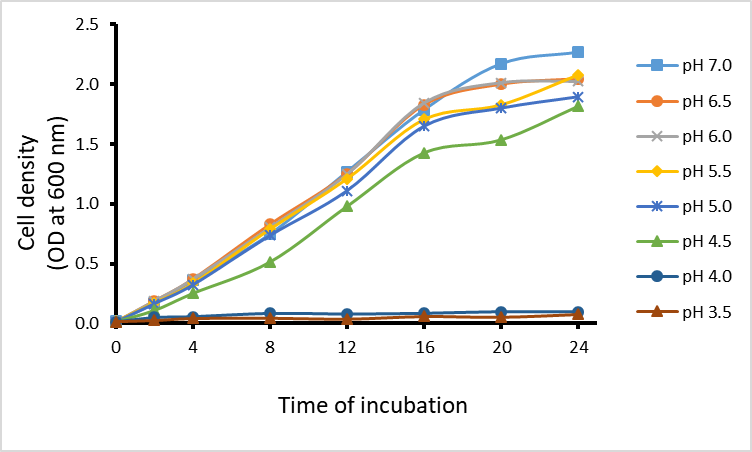
**

**Figure S1.** Growth curve of *Bacillus subtilis* MBB3B9 at different pH. Data represents mean optical density (OD) at 600 nm from three independent replications.


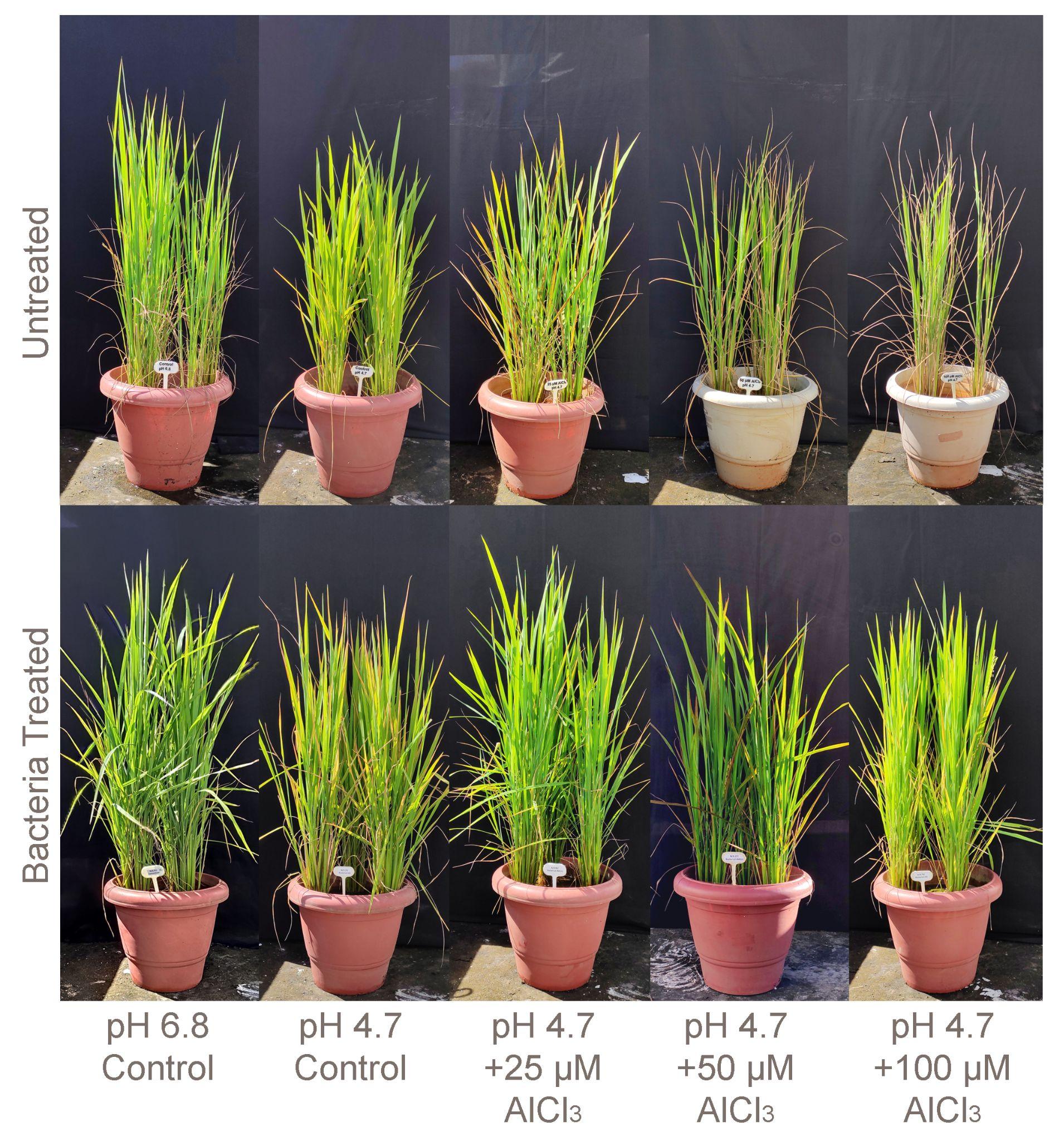


**Figure S2.** Pot experiment showing plant growth in bacteria treated and untreated soil under control and increasing Al-stress conditions.

**Table S1.** Morphological and biochemical properties of *Bacillus subtilis* MBB3B9

| 1. **Morphological Characters** | | | | | | | | |
| --- | --- | --- | --- | --- | --- | --- | --- | --- |
| **Characters** | | | | **Result** | | | | |
| **Colony morphology** | | | | **Pale white, opaque, rough margin, irregular** | | | | |
| **Gram’s reaction** | | | | **Gram positive** | | | | |
| **Cell shape, size** | | | | **Rod shaped, 2-3 µm** | | | | |
| **Sporulation** | | | | **Positive** | | | | |
| **Biofilm formation** | | | | **Positive** | | | | |
| 1. **Biochemical Characters** | | | | | | | | |
| **i) Biochemical Tests using API 50 CHB** | | | | | |  | **ii) Biochemical Tests using API 20** | |
| **Substrate name** | **Result** |  | **Substrate name** | | **Result** |  | **Tests name** | **Result** |
| Glycerol | Positive |  | Salicin | | Positive |  | Catalase | Positive |
| Erythritol | Negative |  | Cellobiose | | Positive |  | ONPG | Positive |
| D-Arabinose | Negative |  | Maltose | | Positive |  | ADH | Negative |
| L-Arabinose | Positive |  | Lactose | | Weak Positive |  | LDC | Negative |
| Ribose | Positive |  | Melibiose | | Weak Positive |  | ODC | Weak Positive |
| D-Xylose | Negative |  | Saccharose | | Positive |  | Citrate | Positive |
| L-Xylose | Negative |  | Trehalose | | Positive |  | H2S | Negative |
| Adonitol | Negative |  | Inulin | | Positive |  | Urease | Negative |
| Β-Methyl-D-xyloside | Positive |  | Melezitose | | Negative |  | TDA | Negative |
| Galactose | Positive |  | D-Raffinose | | Weak Positive |  | Indole | Negative |
| D-Glucose | Positive |  | Amidon | | Weak Positive |  | MR | Negative |
| D-Fructose | Positive |  | Glycogen | | Positive |  | VP | Positive |
| D-Mannose | Negative |  | Xylitol | | Negative |  | Gelatin | Positive |
| L-Sorbose | Negative |  | β-Gentiobiose | | Weak Positive |  | Nitrate | Positive |
| Rhamnose | Negative |  | D-Turanose | | Weak Positive |  | Lechithinase | Positive |
| Dulcitol | Negative |  | D-Lyxose | | Negative |  | Protease | Positive |
| Inositol | W |  | D-Tagatose | | Negative |  |  | |
| Mannitol | Positive |  | D-Fucose | | Negative |  | **iii) Growth at different temperatures (°C)** | |
| Sorbitol | Positive |  |  |  |  |  |  |  |
| Arbutin | Positive |  | L-Fucose | | Negative |  | 4 °C | Negative |
| Methyl- α -D-mannopyranoside | Negative |  | D-Arabitol | | Negative |  | 15 °C | Weak Positive |
| Methyl-α-D-glucopyranoside | Positive |  | L-Arabitol | | Negative |  | 30 °C | Positive |
| N-Acetylglucosamine | W |  | Gluconate | | Negative |  | 40 °C | Positive |
| Amygdalin | Positive |  | 2-Ketogluconate | | Negative |  | 45 °C | Weak Positive |
| Esculin | Weak Positive |  | 5-Ketogluconate | | Negative |  | 50 °C | Negative |

**Table S2.** Description of genes potentially involved in acid stress induced Al-tolerance.

| **PGP properties** | **Gene** | **Enzyme** | **Particular function** | **KEGG pathway** |
| --- | --- | --- | --- | --- |
| Siderophore production | *dhbA*/*entA* | 2,3-dihydro-2,3-dihydroxybenzoate dehydrogenase | Bacillibactin biosynthesis | K00216 |
|  | *dhbB* | bifunctional isochorismate lyase / aryl carrier protein | Bacillibactin biosynthesis | K01252 |
|  | *dhbC* | isochorismate synthase | Bacillibactin biosynthesis | K02361 |
|  | *dhbE* | 2,3-dihydroxybenzoate---[aryl-carrier protein] ligase | Bacillibactin biosynthesis | K02363 |
|  | *dhbF* | glyine---[glycyl-carrier protein] ligase | Bacillibactin biosynthesis | K04780 |
|  | *ymfD* | MFS transporter | Bacillibactin export | K08221 |
| Phosphate solubilization, Potassium solubilization | *frdA* | fumarate reductase, flavoprotein subunit | Succinic Acid Biosynthesis | K00239 |
|  | *ghrB* | glyoxylate/hydroxypyruvate/2-ketogluconate reductase | Keto Gluconate Biosynthesis | K00090 |
|  | *maeA*/*sfcA* | Malate dehydrogenase | Malic/Pyruvic Acid Biosynthesis | K00027 |
|  | *ackA* | Acetate kinase | Propionic Acid Biosynthesis | K00925 |
|  | *pta* | phosphate acetyltransferase | Acetic/Propionic Acid Biosynthesis | K00625 |
|  | *acyP* | acylphosphatase | Acetic Acid Biosynthesis | K01512 |
|  | *lpd*/*pdhD* | dihydrolipoamide dehydrogenase | Acetoin 2,3-butandiol Biosynthesis | K00382 |
|  | *aceF*/*pdhC* | pyruvate dehydrogenase E2 component (dihydrolipoamide acetyltransferase) | Acetoin 2,3-butandiol Biosynthesis | K00627 |
|  | *actP* | cation/acetate symporter | Acetic Acid Transport | K14393 |
|  | *pckA* | phosphoenolpyruvate carboxykinase (ATP) | Oxaloacetic Acid Biosynthesis | K01610 |
|  | *pyc* | pyruvate carboxylase | Oxaloacetic Acid Biosynthesis | K01958 |
|  | *mdh* | malate dehydrogenase | Malic Acid Biosynthesis | K00024 |
|  | *dctA* | aerobic C4-dicarboxylate transport protein | Succinic Acid Transport | K11103 |
|  | *CS*/*gltA* | citrate synthase | Citric Acid Biosynthesis | K01647 |
|  | *citM* | Mg2+/citrate complex secondary transporter | Citric Acid Transport | citM |
|  | *citS* | two-component system, CitB family, sensor histidine kinase CitS | Citric Acid Transport | K11637 |
|  | *sucA* | 2-oxoglutarate dehydrogenase E1 component | Succinic Acid Biosynthesis | K00164 |
|  | *sucB* | 2-oxoglutarate dehydrogenase E2 component | Succinic Acid Biosynthesis | K00658 |
|  | *sucC* | succinyl-CoA synthetase beta subunit | Succinic Acid Biosynthesis | K01903 |
|  | *sucD* | succinyl-CoA synthetase alpha subunit | Succinic Acid Biosynthesis | K01902 |
|  | *prpB* | methylisocitrate lyase | Succinic Acid Biosynthesis | K03417 |
|  | *fadA*/*fadI* | acetyl-CoA acyltransferase | Succinic/Jasmonic Acid Biosynthesis | K00632 |
|  | *gabD* | succinate-semialdehyde dehydrogenase / glutarate-semialdehyde dehydrogenase | Fumaric Acid Biosynthesis | K00135 |
|  | *sdhA*/*frdA* | succinate dehydrogenase /fumarate reductase, flavoprotein subunit | Succinic Acid Biosynthesis | K00239 |
|  | *sdhC*/*frdC* | succinate dehydrogenase / fumarate reductase, cytochrome b subunit | Fumaric Acid Biosynthesis | K00241 |
|  | *sdhB*/*frdB* | succinate dehydrogenase / fumarate reductase, iron-sulfur subunit | Fumaric Acid Biosynthesis | K00240 |
|  | *fumC* | fumarate hydratase, class II | Malic Acid Biosynthesis | K01679 |
|  | *accA* | acetyl-CoA carboxylase carboxyl transferase subunit alpha | Malonic Acid Biosynthesis | K01962 |
|  | *accB* | acetyl-CoA carboxylase biotin carboxyl carrier protein | Malonic Acid Biosynthesis | K02160 |
|  | *accC* | acetyl-CoA carboxylase, biotin carboxylase subunit | Malonic Acid Biosynthesis | K01961 |
|  | *accD* | acetyl-CoA carboxylase carboxyl transferase subunit beta | Malonic Acid Biosynthesis | K01963 |
|  | *pccB* | propionyl-CoA carboxylase beta chain | Malonic Acid Biosynthesis | K01966 |
|  | *ldh* | L-lactate dehydrogenase | Lactic Acid Biosynthesis | K00016 |
|  | *gloB*/*gloC* | hydroxyacylglutathione hydrolase | Lactic Acid Biosynthesis | K01069 |
|  | *mgsA* | methylglyoxal synthase | Lactic Acid Biosynthesis | K01734 |
|  | *lctP* | lactate permease | Lactic Acid Transport | K03303 |
|  | *ptb* | phosphate butyryltransferase | Butyric Acid Biosynthesis | K00634 |
|  | *buk* | butyrate kinase | Butyric Acid Biosynthesis | K00929 |
|  | *ybgC* | acyl-CoA thioester hydrolase | Valeric Acid Biosynthesis | K07107 |
|  | *ilvD* | dihydroxy-acid dehydratase | Pyruvic Acid Biosynthesis | K01687 |
|  | *dat* | D-alanine transaminase | Pyruvic Acid Biosynthesis | K00824 |
|  | *tpa* | taurine-pyruvate aminotransferase | Pyruvic Acid Biosynthesis | K03851 |
|  | *ilvA*/*tdcB* | threonine dehydratase | Pyruvic Acid Biosynthesis | K01754 |
|  | *dsdA* | D-serine dehydratase | Pyruvic Acid Biosynthesis | K01753 |
|  | *sdaA*/*sdaB*/*tdcG* | L-serine dehydratase | Pyruvic Acid Biosynthesis | K01752 |
|  | *patB* | cysteine-S-conjugate beta-lyase | Pyruvic Acid Biosynthesis | K14155 |
|  | *pyk* | pyruvate kinase | Pyruvic Acid Biosynthesis | K00873 |
|  | *acs* | acetyl-CoA synthetase | Propionic Acid Biosynthesis | K01895 |
|  | *acuI* | acrylyl-CoA reductase | Propionic Acid Biosynthesis | K19745 |
|  | *mmsA*/*iolA* | methylmalonate-semialdehyde dehydrogenase | Propionic Acid Biosynthesis | K00140 |
|  | *mmdA* | methylmalonyl-CoA decarboxylase subunit alpha | Propionic Acid Biosynthesis | K01604 |
|  | *ttuB* | MFS transporter, ACS family, tartrate transporter | Tartaric Acid Transport | K13021 |
|  | *phnO* | Uncharacterized N-acetyltransferase | Phosphonate degradation |  |
|  | *phoA* | Alkaline phosphatase | Alkaline Phosphatase Activity | K01077 |
|  | *phoD* | Alkaline phosphatase | Alkaline Phosphatase Activity | K01113 |
|  | *phy* | 3-phytase | Phytase Production | K01083 |
|  | *phoE* | uncharacterized phosphatase | Phosphatase Activity | K15640 |
|  | *pstA* | phosphate transport system permease protein | Phosphate Transport | K02038 |
|  | *pstB* | phosphate transport system ATP-binding protein | Phosphate Transport | K02036 |
|  | *pstC* | phosphate transport system permease protein | Phosphate Transport | K02037 |
| Nitrogen metabolism | *nifF* | flavodoxin I | Nitrogenase Biosynthesis | K03839 |
|  | *nifU* | nitrogen fixation protein NifU | Nitrogenase Biosynthesis | K04488 |
|  | *narG*/*narZ*/*nxrA* | nitrate reductase / nitrite oxidoreductase, alpha subunit | Denitrification | K00370 |
|  | *nfrA1* | FMN reductase (NADPH) | Denitrification | K19285 |
|  | *nfrA2*/*ycnD* | FMN reductase [NAD(P)H] | Denitrification | K19286 |
|  | *narK*/*nrtP*/*nrt*/*narU* | MFS transporter, NNP family, nitrate/nitrite transporter | Denitrification | K02575 |
|  | *norQ* | nitric oxide reductase NorQ protein | Denitrification |  |
|  | *nasA* | assimilatory nitrate reductase catalytic subunit | Denitrification | K00372 |
|  | *nirB* | nitrite reductase (NADH) large subunit [EC:1.7.1.15] | Denitrification | K00362 |
|  | *narH*/*narY*/*nxrB* | nitrate reductase / nitrite oxidoreductase, beta subunit | Denitrification | K00371 |
|  | *narI*/*narV* | nitrate reductase gamma subunit | Denitrification | K00374 |
|  | *narJ*/*narW* | nitrate reductase molybdenum cofactor assembly chaperone NarJ/NarW | Denitrification | K00373 |
|  | *nirC* | nitrite transporter | Denitrification | K02598 |
|  | *gltB* | glutamate synthase (NADPH) large chain | Glutamate/Glutamine Metabolism | K00265 |
|  | *gltD* | glutamate synthase (NADPH) small chain | Glutamate/Glutamine Metabolism | K00266 |
|  | *glnA* | glutamine synthetase | Glutamate/Glutamine Metabolism | K01915 |
|  | *glnB* | nitrogen regulatory protein P-II | Glutamate/Glutamine Metabolism | K04751 |
|  | *peb1A*/*glnH* | aspartate/glutamate/glutamine transport system substrate-binding protein | Glutamine Transport | K10039 |
|  | *peb1C/glnQ* | aspartate/glutamate/glutamine transport system | Glutamine Transport | K10041 |
|  | *peb1B/glnP/glnM* | aspartate/glutamate/glutamine transport system permease protein | Glutamine Transport | K10040 |
|  | *glnK* | two-component system, sensor histidine kinase GlnK | Glutamine Transport | K07717 |
|  | *glnL* | two-component system, response regulator GlnL | Glutamine Transport | K07719 |
|  | *glnT* | putative sodium/glutamine symporter | Glutamine Transport | K11626 |
|  | *gltP*/*gltT* | proton glutamate symport protein | Glutamate Transport | K11102 |
|  | *ureA* | urease subunit gamma | Urea Metabolism | K01430 |
|  | *ureB* | urease subunit beta | Urea Metabolism | K01429 |
|  | *ureC* | urease subunit alpha | Urea Metabolism | K01428 |
| Auxin Production | *pyc* | pyruvate carboxylase | Indole 3-Acetic Acid Biosynthesis | K01958 |
|  | *trpA* | tryptophan synthase alpha chain | Tryptophn Biosynthesis | K01695 |
|  | *trpB* | tryptophan synthase beta chain | Tryptophn Biosynthesis | K01696 |
|  | *trpC* | indole-3-glycerol phosphate synthase | Tryptophn Biosynthesis | K01609 |
|  | *trpD* | anthranilate phosphoribosyltransferase | Tryptophn Biosynthesis | K00766 |
|  | *trpE* | anthranilate synthase component I | Tryptophn Biosynthesis | K01657 |
|  | *bsdC* | vanillate/4-hydroxybenzoate decarboxylase subunit C | Indole 3-Acetic Acid Biosynthesis | K01612 |
|  | *aldH* | aldehyde dehydrogenase (NAD+) | Indole 3-Acetic Acid Biosynthesis | K00128 |
|  | *patA* | aminotransferase | Indole 3-Acetic Acid Biosynthesis | K00841 |
|  | *poxL* | pyruvate oxidase | Indole 3-Acetic Acid Biosynthesis | K00158 |
|  | *yedL* | putative acetyltransferase | Indole 3-Acetic Acid Biosynthesis | K03829 |
| Cytokinin production | *miaA*/*ipt* | tRNA dimethylallyltransferase | Cytokinin Biosynthesis | K00791 |
|  | *miaB* | tRNA-2-methylthio-N6-dimethylallyladenosine synthase | Cytokinin Biosynthesis | K06168 |
|  | *dapF* | diaminopimelate epimerase | Cytokinin Biosynthesis | K01778 |
|  | *xdhA* | dehydrogenase molybdenum-binding subunit | Xanthine Biosynthesis | K00087 |
|  | *xdhB* | xanthine dehydrogenase FAD-binding subunit | Xanthine Biosynthesis | K13479 |
|  | *xdhC* | xanthine dehydrogenase accessory factor | Xanthine Biosynthesis | K07402 |

Table S3: Comparative analysis of potential virulent factors in the genomes of *B. subtilis* MBB3B9 and *B. subtilis* MZK05.

| Locus tag | | Gene name | Functional protein | Category |
| --- | --- | --- | --- | --- |
| *B. subtilis* MBB3B9 | *B. subtilis* MZK05 |  |  |  |
| LSG27_02585 | D3Z17_20725 | hypothetical | T7SS effector LXG polymorphic toxin | Virulence factor-toxin (effector protein) |
| LSG27_03845 | D3Z17_17785 | hypothetical | NisI/SpaI family lantibiotic immunity lipoprotein | Virulence factor- lantibiotic immunity lipoprotein |
| LSG27_05275 | D3Z17_16350 | bslA | biofilm surface layer hydrophobin BslA | Virulence factor- adhesion protein |
| LSG27_06830 | D3Z17_14775 | clpX | ATP-dependent protease ATP-binding subunit ClpX | Virulence factor-protease |
| LSG27_08080 | D3Z17_13555 | bsrH | type I toxin-antitoxin system toxin BsrH | Virulence factor-t7SS immunity protein |
| LSG27_08085 | D3Z17_13550 | txpA | type I toxin-antitoxin system toxin BsrH | Virulence factor-t7SS immunity protein |
| LSG27_08185 | D3Z17_13455 | yqcF | type VII secretion system immunity protein YqcF | Virulence factor-toxin (effector protein) |
| LSG27_08190 | D3Z17_13450 | yqcG | LXG family T7SS effector endonuclease toxin YqcG | Virulence factor-toxin (effector protein) |
| LSG27_12635 | full length gene not found | bsrG | type I toxin-antitoxin system toxin BsrG | Virulence factor-t7SS immunity protein |
| LSG27_12975 | D3Z17_08735 | bcbE | bacillibactin exporter BcbE | Virulence factor- bacillibactin export |
| LSG27_13330 | D3Z17_8380 | codY | GTP-sensing pleiotropic transcriptional regulator CodY | Virulence factor- transcriptional repressor |
| LSG27_19270 | D3Z17_02385 | ndoA | type II toxin-antitoxin system endoribonuclease NdoA | Virulence factor-endonuclease |
